# Molecular Investigation of the Fatal Bloodstream *Candida orthopsilosis* Infection Case following Gastrectomy

**DOI:** 10.3390/ijms24076541

**Published:** 2023-03-31

**Authors:** Magdalena Mnichowska-Polanowska, Magdalena Adamowicz, Iwona Wojciechowska-Koszko, Anna Kisiel, Bartosz Wojciuk, Konrad Jarosz, Barbara Dołęgowska

**Affiliations:** 1 Department of Medical Microbiology, Immunology and Laboratory Medicine, Pomeranian Medical University, 70-204 Szczecin, Poland; 2 Clinic of Anaesthesiology and Intensive Therapy, Teaching Hospital No 1, Pomeranian Medical University, 70-204 Szczecin, Poland; 3 Institute of Marine and Environmental Sciences, University of Szczecin, 70-383 Szczecin, Poland

**Keywords:** antifungals, *Candida orthopsilosis*, CBSI, candidemia

## Abstract

*Candida orthopsilosis* represents a closely related cryptic genospecies of *Candida parapsilosis* complex-misidentified in routine diagnostic assays. This is emerging in settings where central venous catheters, invasive medical interventions, and echinocandin treatments are most likely to be used. A 59-year-old, non-neutropenic male patient, was admitted to an intensive care unit (ICU) due to respiratory distress syndrome, following a partial gastrectomy. As a result of duodenal stump leakage, re-laparotomy was required, abdominal drains were provided and central line catheters were exchanged. Multiple isolates of *Candida orthopsilosis* drawn from consecutive blood cultures were identified, despite ongoing echinocandin therapy and confirmed in vitro echinocandins susceptibility of the isolated strain. Species identification was verified via ITS region sequencing. Herein, we report the well-documented—per clinical data and relevant laboratory diagnosis—first case of a bloodstream infection caused by *Candida orthopsilosis* in Poland.

## 1. Introduction

ICU-associated CBSIs (Candida bloodstream infections) in adults, characterized by a high mortality rate (42–85%) [1,2,3], are commonly caused by the *Candida (C.)* non-albicans group [4], in which *Candida (C.) parapsilosis* appears to be increasing and is probably due to overuse of echinocandins [5,6,7,8,9]. This species has been reported as the second or third most prevalent agent of ICU-acquired CBSI in South East Asia, Latin America (Brasil and Chile), refs. [10,11,12] and Southern and Central Europe (Italy, Spain, and Poland) [3,13,14,15]. Although published papers on ICU-associated CBSIs in Poland are scarce, *C. parapsilosis* has been ranked as the second cause of CBSI [15]. In fact, the *C. parapsilosis* complex species is a heterogenous group composed of the following closely related cryptic genospecies: *C. parapsilosis* sensu stricto, *Candida (C.) metapsilosis*, and *Candida (C.) orthopsilosis* [16]. *C. orthopsilosis* first emerged in 2005 as a potential human cryptic pathogen and remains a relatively uncommon aethiological agent of CBSIs with an unknown 30-day mortality rate [17,18,19,20,21]. *C. orthopsilosis* is found in patients with prolonged use of central venous catheters, previous surgical history, and invasive abdominal interventions with total parenteral nutrition [17,19]. The prevalence and transmission of *C. orthopsilosis*-caused BSI in ICUs varies geographically and ranges from 1.0 to 28.3% [17,18,20,22].

Epidemiological data are underestimated because broadly implemented biochemical identification systems (VITEK-2 Compact and BD Phoenix) frequently misidentify *C. orthopsilosis*. The use of diagnostic assays is not highly efficient in cryptic species detection, which results in both delayed diagnosis and delayed antifungal therapy of CBSI. Consequently, this is challenging patient’s survival. Nowadays, mass spectrometry, fungal ITS DNA–barcoding, or automated molecular assays, are required for the effective detection of uncommon *C. orthopsilosis* genospecies [23,24]. More data with a combined clinical and molecular approach are needed to explain the uncertainty regarding practical implications of *C. orthopsilosis* identification. On this basis, we herein describe the well-documented case of bloodstream infection caused by *C. orthopsilosis* in the ICU which originates from our single-centre prospective surveillance of invasive fungal infections in the ICU. To our knowledge, this is the first CBSI case from Poland caused by *C. orthopsilosis*, as described in this paper.

## 2. Case Presentation

A non-neutropenic, 59-year-old male patient, was admitted to an ICU at University Hospital in Szczecin, Poland due to shortness of breath and respiratory distress that developed following a partial gastrectomy. The gastrectomy was a result of previous gastric ulcer perforation.

On ICU admission, the patient’s height and weight were 180 cm and 90 kg (body mass index (BMI): 27.8), respectively. His respirations were unstable and mechanical ventilation with oxygen of 40% was administered. Blood pressure of 110/70 mmHg, heart rate of 95 beats/min with regular rhythm, and body temperature of 38.2 °C were observed. Laboratory parameters on ICU admission were the following: elevated white blood cell count (WBC) of 29,000 cells/mm^3^ (reference range, 4000 to 10,000 cells/mm^3^), and elevated C-reactive protein (CRP) level of 232 mg/l (reference range, <5 mg/dL), in combination with low procalcitonin level (PRC)—0.6 ng/mL (reference range 0.5–2 ng/mL). Due to pneumonia, diagnosed by chest X-ray, the intravenous empiric therapy with piperacillin/tazobactam (each dose of 4.5 g at a 6 h interval) was applied and maintained for 9 days. Mortality risk ratios assessed with SOFA (Sepsis-related Organ Failure Assessment) and APACHE II (Acute Physiology and Chronic Health Evaluation) scales were 15–20% (8 points) and 29% (18 points), respectively. Fungal colonization had been screened by culturing samples collected from commonly colonized body sites to predict the *Candida* colonization index (CI). The anal, urine, and bronchoalveolar samples were negative for *Candida*, whereas swabs from underarm skin, groin skin, and the nasal cavity, were positive for *C. parapsilosis* sensu stricto. Confirmed multifocal colonization and clinical parameters, such as parenteral nutrition and post-abdominal surgery, resulted in a Candida Score (CS) index ≥ 3. Following CS index and ESCMID (European Society of Clinical Microbiology and Infectious Diseases) guidelines [25], fluconazole prophylaxis was introduced (initial day—400 mg divided into two doses of 200 mg at an interval of 12 h; following days—200 mg each at an interval of 24 h) and continued for 7 days.

On the 8th day, fluconazole was replaced with micafungin (each dose equal to 100 mg/day) as a result of deterioration of the patient’s general condition, acute abdominal symptoms, and leakage of intestinal contents through abdominal drains. Due to leakage from the duodenal stump, urgent re-laparotomy was required. After total gastrectomy with esophago-intestinal anastomosis, the wound was stitched, and abdominal drains were provided.

On the 11th day, empiric broad spectral antibacterial therapy with imipenem and vancomycin was provided due to increasing inflammatory parameters (CRP—436 mg/L, PRC—0.7 ng/mL). Multiple blood cultures remained negative and micafungin was continued.

On the 11th and 13th day, two re-relaparotomies accompanied by rinsing of the peritoneal cavity—while leaving the abdominal cavity open—were performed. The patient received total parenteral nutrition as a result of ineffective enteral nutrition previously provided by the Flocar probe, inserted behind Treitz ligament; a leakage of the administered glucose through the open abdominal wall was observed. No microorganisms were cultivated from the blood samples at this stage while laboratory indicators of infection were persistently high (WBC—29,000 cells/mm^3^, CRP—513 mg/L, PRC—1.03 ng/mL).

On the 13th day, *Candida*–bloodstream infection (CBSI) was established through multiple isolations of *C. orthopsilosis* from blood cultures with confirmed susceptibility to all echinocandins [26]. Despite micafungin administration, over the following consecutive days of the patient’s ICU stay, *C. orthopsilosis* was isolated from peripheral blood and from blood samples collected via the central line catheter. The suspected catheter was removed and submitted to MAKI culture technique to determine the risk of CLABSI (central line associated BSI). Following MAKI testing, *C. orthopsilosis* was detected and all possible catheters, as well as abdominal drains, were exchanged. According to the microbiological report, micafungin had been continued [25,27,28]. Unfortunately, no further surgical treatment was available, although gastrointestinal leakage was observed. On the 40th day of the ICU stay, the patient died without resolution of CBSI.

### 2.1. Early Monitoring of the Risk of CBSI by Fungal Cell Wall Biomarkers

Testing of mannan and 1,3 β-D-glucan for early CBSI detection revealed conflicting data. Weekly tested serum mannan antigen was negative with concentrations below the cut-off point for positive samples of <62.5 pg/mL; whereas 1,3 β-D-glucan values were high above the cut-off threshold of 80 pg/mL, ranging from 212.3 to 606.0 pg/mL. Additionally, 1,3 β-D-glucan was detected one day before CBSI diagnosis was established via positive blood culture. Decreasing 1,3 β-D-glucan concentrations were noted after micafungin administration (Table 1).

### 2.2. Mass Spectrometry Biotyping of C. orthopsilosis Isolates

**Figure 1:**
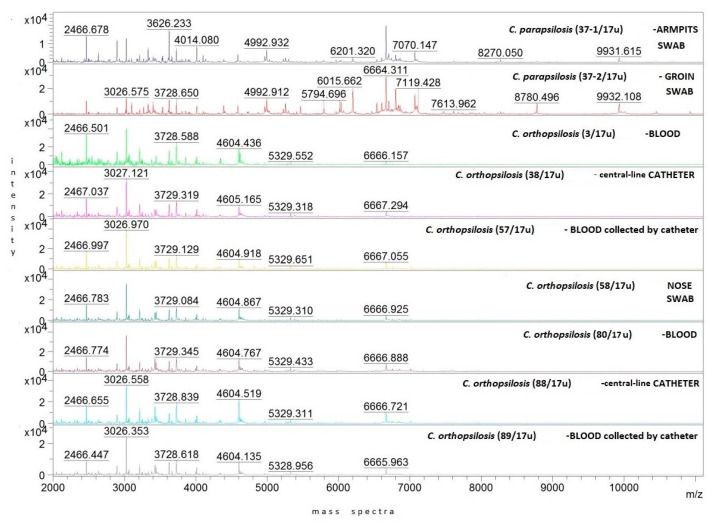
Representative MSPs for clinical *C. parapsilosis* and *C. orthopsilosis* strains isolated from different body locations of 59-year-old male patient with CBSI. Mass spectra intensity displayed in arbitrary units (a.u. = 0, 1, 2, 3, 4 × 10^4^) on the y-axis, whereas mass-to-charge ratio expressed in m/z units on the x-axis; color was assigned to number of strain.

**Figure 2:**
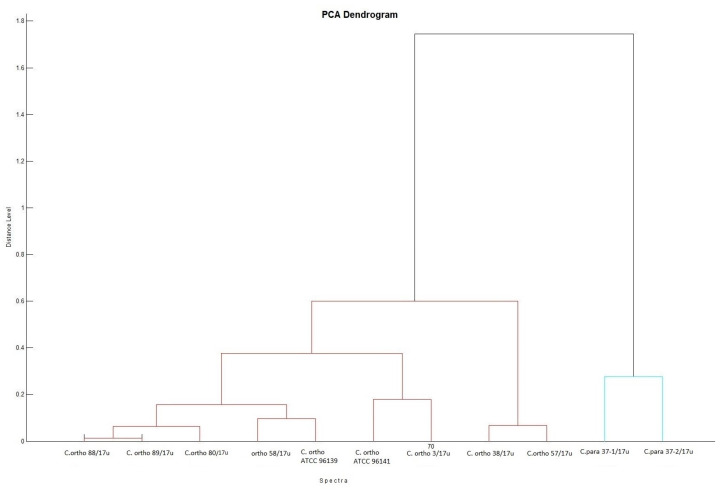
Biotyper PCA (principal component analysis) dendrogram clustering representative MSPs for isolates of *C. orthopsilosis* and *C. parapsilosis* with distances displayed in relative units on the y-axis; MSPs for isolates of *C. orthopsilosis* are clustered with MSPs for reference *C. orthopsilosis* ATCC 96139 and ATCC 96141. Protein similarity presented on dendrogram by means of a relative distance level between isolates included into dendrogram, normalized to a maximum value of 1000 (cut-off). All tested isolates are presented by their abbreviated species name (*C*. *ortho* and *C*. *para*) and the isolation number.

Representative protein mass spectra (MSPs) for all particular isolates are presented in Figure 1. Both *C. orthopsilosis* and *C. parapsilosis* isolates have been grouped based on their protein MSPs into two clusters (Figure 2). *C. orthopsilosis* has been misidentified as *C. parapilosis* or *C. famata* by biochemical platform VITEK-2 Compact.

### 2.3. Antifungal Susceptibility of C. orthopsilosis Isolates

The minimum concentration of antifungals required to inhibit *C. parapsilosis* and *C. orthopsilosis* growth was determined by E-test gradient strips and the automated VITEK-2 system. Skin-colonizing isolates of *C. parapsilosis* were susceptible to amphotericin B (MIC = 0.03 µg/mL), fluconazole (MIC = 0.38 µg/mL), and echinocandins (MIC range 0.25 to 0.50 µg/mL). The cryptic isolates of *C. orthopsilosis* were also susceptible to amphotericin B (MIC range 0.03 to 0.25 µg/mL), fluconazole (MIC range 0.19 to 0.50 µg/mL), and all echinocandins—caspofungin (max MIC = 0.50 µg/mL), micafungin (max MIC = 1.00 µg/mL), and anidulafungin (max MIC = 0.50 µg/mL). EUCAST MIC breakpoints and epidemiologic cut-off (ECOFF) values for *C. orthopsilosis* are still not established in the updated EUCAST guidelines [20], therefore MIC breakpoints for *C. parapsilosis* were applied to determine antifungal resistance for *C. orthopsilosis*. Multiple isolates of *C. orthopsilosis*, from blood and the nasal cavity, were similar in MIC values against echinocandins when compared with reference *C. orthopsilosis* strain ATCC 96141. *C. orthopsilosis* strains isolated from catheter tips and from blood collected by catheters had elevated MICs (MIC = 1.0 µg/mL and MIC = 0.75 µg/mL respectively) against micafungin but were still susceptible to this drug, according to EUCAST recommendations (Table 2).

### 2.4. ITS Sequencing of C. orthopsilosis Isolates

**Figure 3:**
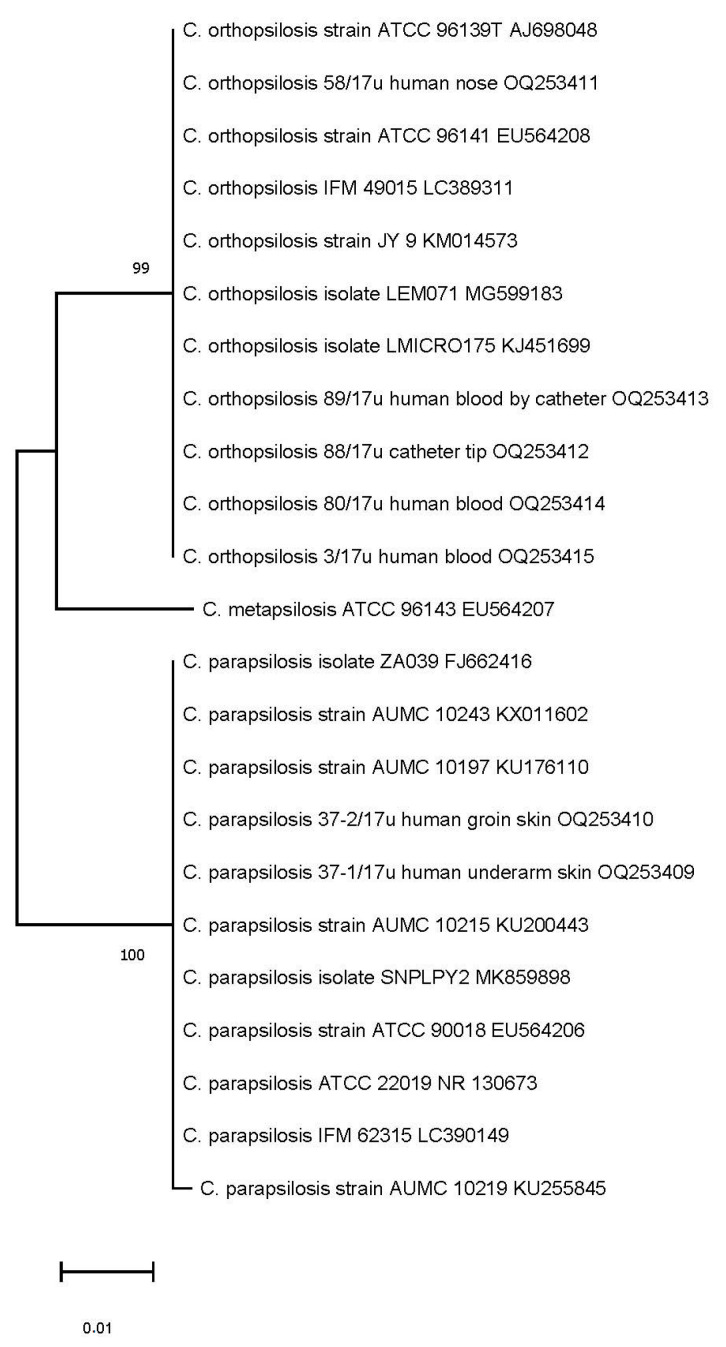
Phylogenetic neighbour-joining tree reflecting a genetic similarity of ITS sequences. Numbers at each branch indicate similarity percentage, expressed in bootstrap values of replicate trees in which the associated taxa clustered together in the bootstrap test (1000 replicates). The scale bar indicates 0.01 changes/site (number of differences).

The dendrogram based on the shared ITS sequence similarity revealed three clusters for *C. orthopsilosis, C. parapsilosis* sensu stricto, and *C. metapsilosis* (ATCC 96143), when included as a mixture of isolates for three genospecies. Our isolates with a high ITS homology were clustered into two well-differentiated groups, assigned to *C. orthopsilosis* and *C. parapsilosis* sensu stricto by neighbour-joining analysis (Figure 3). The ITS region sequences for *C. orthopsilosis* were 96% similar to *C. parapsilosis* sequences and >99% similar to *C. orthopsilosis* sequences of blood isolates found in NCBI GenBank. Our blood and catheter isolates from the hospital in Poland were closely related (99.80–100% ITS match) to blood (ATCC 96141) and catheter (ATCC 96139T) strains isolated in San Antonio, TX, USA. A 100% similarity in ITS region between *C. orthopsilosis* isolated from blood and central line collected blood from our patient can suggest central line associated origin of CBSI. *C. orthopsilosis* blood isolates derived from different timepoint collections were also highly similar when tested via ITS sequencing. As revealed by ITS sequencing, isolates from skin were assigned to *C. parapsilosis* sensu stricto and were not detected in other body sites in addition to the patient’s blood samples during his hospital stay. ITS sequences, determined in the study for complex genospecies, were deposited in GenBank with the accession numbers shown in Table 3 (column 3 from left side).

### 2.5. Materials and Methods

#### 2.5.1. Blood Culturing and Species Identification

Multiple blood samples were collected during every episode of fever for culturing in automated microbiological system BacT/ALERT 3D (bioMérieux, Warsaw, Poland). Species identification was performed on the day of indicated yeast growth by means of automated multiplex PCR (Film Array BCID Panel, bioMérieux, Warsaw, Poland). *C. orthopsilosis* was off—assay panel and finally was not identified within this system. All *Candida* strains isolated from the patient were identified by VITEK-2 system (bioMérieux, Warsaw, Poland) as *C. parapsilosis* or *C. famata*. MALDI-Biotyper (Bruker Daltonics, Bremen, Germany) re-classified the samples from blood, central line catheter, and nasal as *C. orthopsilosis* with high confidence score value > 2. Each isolate tested six times to submit the high-confidence mass signatures to mass spectrometry strain biotyping. Unique cell-wall components of yeast: mannan (Bio-Rad, Warsaw, Poland) and 1,3 β-D-glucan (Fungitell, Associates of Cape Cod, East Falmouth, MA, USA) were assessed simultaneously directly in serum samples.

#### 2.5.2. Genetic Analysis

ITS region sequencing was used to verify the outcomes provided by MALDI–Biotyper. Preceding genomic DNA extraction was made using a commercial DNA extraction kit (EURx, Gdańsk, Poland) with several of our own modifications. Preliminarily, the yeasts were incubated for 20 h on Sabouraud dextrose agar at 30 °C. Subsequently, these were then washed, frozen in liquid nitrogen, and crushed via a grinding tool (EURx, Gdansk, Poland). Implementing the same method used for fungal genomic DNA extraction, we directly extracted from *Candida*-positive blood samples. The DNA amplification was performed with universal primers ITS1 (5’-TCCGTAGGTGAACCTGCGG-3’) and ITS4 (5’-TCCTCCGCTTATTGATATGC-3’) (Genomed, Warsaw, Poland), and revealed the PCR product corresponding to the ITS1–5.8S–ITS2 rDNA region (shortly ITS). The PCR was carried out with the same conditions as previously described [21,29]; amplicons were then purified and sequenced by Sanger DNA sequencing in both the forward and reverse directions (Genomed, Warsaw, Poland) for molecular identification of Candida species.

#### 2.5.3. Bioinformatic Analysis

ITS nucleotide sequences of particular isolates that were revealed in the study were uploaded to BLAST analysis (http://blast.ncbi.nlm.nih.gov (accessed on 27 February 2023). They were compared with ITS sequences, available in NCBI GenBank, for the reference species of *C. orthopsilosis* (ATCC 96141 and ATCC 96139T), *C. parapsilosis* (ATCC 90018 and ATCC 22019), as well as for blood and catheter isolates derived from CBSI-derived samples. Then, those have been submitted to multiple ITS sequence alignments with ClustalW algorithm in Mega11 online software. The aligned sequences (Figure S1 were processed, and the phylogenetic tree as a dendrogram based on the neighbour-joining method was generated, reflecting the percentage of ITS sequence similarity with 1000 bootstrap replicates.

## 3. Discussion

This report is focused on a clinical and diagnosis approach to discuss the fatal case of *C. orthopsilosis* BSI. Nowadays, the *C. orthopsilosis* and cryptic non-albicans species are changing the epidemiology of CBSI worldwide and the overall incidence of CBSI, due to the cryptic species, seems to be increasing [17,22,23]. Although, there are still limited, underrated data on *C. orthopsilosis* as a causative agent for CBSI in the ICU [8,17]. Clinical studies focused on CBSI in ICU are mostly retrospective and do not discriminate between closely related species [1,3,4]. Simultaneously, laboratory studies mostly skip the clinical implications of the data presented [22]. Improved species identification seems to be crucial to monitor local epidemiology, implement infection control measures, and manage appropriate antifungal therapy [4,30].

In our study, *C. orthopsilosis* has been misidentified as *C. parapsilosis* or *C. famata* by biochemical identification, whereas a mass spectrometry met the daily needs of clinicians as a reliable, cost- and time-effective platform for detection of *C. orthopsilosis* via blood cultures from the ICU patient [30,31]. *C. orthopsilosis* and *C. parapsilosis* shared the epidemiological relationship at ICU; therefore, intraspecies identification with sequencing and matching of ITS region was essential [22,23,32]. The high ITS homology between *C. orthopsilosis* isolated from blood, central line collected blood, and central line catheter, after its removal at the same timepoint, was indicated. Since *C. orthopsilosis* has been recovered from intravascular catheters we concluded that the catheter was the most probable site through which *C. orthopsilosis* reached the bloodstream [22,33]. Previous findings [14] reported that the source of *C. parapsilosis* CBSI was a vascular catheter in more than 50% of cases.

*C. orthopsilosis* has not been found as the patient’s commensal fungus prior to the BSI episode; therefore, a per catheter skin colonization has not been strictly associated with the etiology of catheter related CBSI. This was in accordance with other reports [34] in which the blood isolate belonged to a species other than that of the colonizing site in 13% of the cases. The *C. orthopsilosis* strain, identical to the ones of blood origin, was also found in a nasal swab after CBSI diagnosis, thus a colonization of the patient’s nasal cavity, and a potential strain translocation (by nasogastric tube), cannot be excluded. In-depth analysis of the *C. orthopsilosis* origin on the patient’s catheters can suggest that this cryptic species, similarly to *C. parapsilosis* sensu stricto, is associated with exogenous transmission during catheter care in the respective ICU environment [5,14,23,35].

All *C. orthopsilosis* grouped by ITS neighbour-joining in the same clade with strains isolated in San Antonio, TX, USA (ATCC 96141 and ATCC 96139) could suggest that our isolates represent alternative type 1 subspecies of *C. orthopsilosis* [21,36]. Conversely, as reported by genomic analysis [37], strain ATCC 96141 (MCO456), although closely related to our isolates, when tested by ITS, was a hybrid between two subspecies represented by type 1 and type 2. The relationship between our *C. orthopsilosis* and these clinical isolates from other geographical locations could be explained by advanced metagenomic analysis [37,38,39]. Serological investigation of fungal antigens has been implemented in our study as well and the fungal cell wall components 1,3 β-D-glucan and mannan have been determined. Mannan antigenemia is commonly determined, however 1,3 β-D-glucan assay is now also available in Poland. Simultaneous labelling and monitoring of different biomarkers is considered to increase the sensitivity of testing. In this study, 1,3 β-D-glucan has been detected as a predictor of CBSI while mannan assay showed to be of lower usefulness during the course of *C. orthopsilosis* BSI. Increased 1,3 β-D-glucan content and significantly less mannan were observed for *C. parapsilosis* and *C. orthopsilosis* [40]. This could explain why mannan antigenemia was not detected, despite positive blood cultures. A previous study [41] has shown that the sensitivity of mannan detection in *C. parapsilosis* BSI is low, thus our report corresponds with this finding. *C. orthopsilosis* is considered to be naturally less susceptible to echinocandins, when compared to other non-albicans species [8,42]. A decreased susceptibility is especially presented for micafungin as a result of an intrinsic polymorphism in the Fks1 gene [42]. Thus, the emerging *C. orthopsilosis,* shown in this study, might have expanded by selection resulting from micafungin usage in extended empirical therapy of CBSI. Increased echinocandin usage and its correlation with increased prevalence of BSI caused by *C. parapsilosis* has already been reported [6]. Therefore, we hypothesize that pressure of micafungin–mediated therapy could lead to a selection toward *C. orthopsilosis* and a generation of isolates with elevated values of MICs as well [8,43]. As observed in this study, *C. orthopsilosis* with the higher MICs of micafungin (0.75–1.00 µg/µL) have been isolated from the central line catheter and from the blood collected by the catheter. In our opinion, similarly to other authors [8,43], micafungin–adapted isolates with elevated MIC values, especially on the surface of intravascular catheters, need to be recorded and it also points to the need for increased care of those catheters in ICUs. Kartsonis et al., [44] proved that *C. parapsilosis*- infected patients respond well to standard caspofungin therapy when MIC values for *C. parapsilosis* are 0.50 µg/µL. Another trial [45] revealed that a standard micafungin dosing regimen (100 mg daily) was more effective in therapy of *C. parapsilosis*—mediated candidemia (75.9%) when compared with caspofungin (64.3%). These data support an assumption of potential activity of echinocandins against *C. parapsilosis*. Nevertheless, the standard doses of micafungin were sufficient to overcome the higher MICs of *C. parapsilosis* [45], but it was not sufficient against *C. orthopsilosis.* This cryptic species appeared in the patient’s samples and was persistently isolated from blood. Data regarding the activity of echinocandins against *C. orthopsilosis* are still limited and contradictory. So far, the results of antifungal susceptibility testing to echinocandins, if available, have not been commonly implemented into clinical practice, due to the lack of a clear relationship between the in vitro determined MIC value and the clinical outcome [44,46]. The authors of this study postulate that as long as *C. orthopsilosis* and its antifungal susceptibility data are not included in the epidemiology of CBSI, it will not be possible to obtain comprehensive data to optimize the proper management of CBSI. This may also apply to other crypto species.

Moreover, some ICU patients may not benefit from micafungin therapy due to multiple concomitant risk factors which could impede the success of CBSI treatment [47,48]. High APACHE II scores predicted increased mortality, whereas other risk factors such as the necessity to maintain both central venous catheters and post-surgery abdominal drains, corresponded with factors that have been of great importance in the persistence of infection [49]. Central line catheters infected with *C. orthopsilosis* could be a potential niche for biofilm formation occurring in higher MIC of *C. orthopsilosis* to echinocandin. Echinocandins have been proposed as effective against biofilm as they inhibit polysaccharide production and consequently reduce extracellular matrix [50], whereas other studies have shown antifungal therapy as rarely successful without catheter and drain removals [25,47]. In our study, all catheters had been changed but there was still a need to maintain the access for parenteral nutrition, in addition to catecholamine administration. Consequently, micafungin therapy appeared to be unsuccessful.

In summary, *C. orthopsilosis* was isolated and multiplied in a primarily pneumonic ICU patient, with repeated abdominal surgery, and deteriorating condition. The isolates were obtained via peripheral blood as well as catheter obtained samples. Proper pathogen identification required in-depth analysis as current diagnostic assays remain incapable in distinguishing between most recently reported *Candida* genospecies. In contrast to mannan antigenemia, 1,3 β-D-glucan levels corresponded with microbiological diagnostic outcomes. Precise guidelines to assess *C. orthopsilosis* drug-susceptibility remain unavailable and the patient was treated with micafungin according to the most valid version of EUCAST recommendations dedicated to *C. parapsilosis.* Interestingly, despite genetic identity, MIC values for echinocandins differed in strains isolated from the bloodstream and catheter. This issue requires further molecular studies. Despite potential micafungin activity against *C. orthopsilosis* and observed decrease in 1,3 β-D-glucan concentration, clinical outcome of the treatment appeared poor. Discrepancies between in vitro data on *C. orthopsilosis* drug susceptibility and the clinical outcomes have already been reported and possibly results from other risk factors overlapping in an intensive care patient. The exact origin of the isolated *C. orthopsilosis* strain remains unknown.

## 4. Conclusions

The investigation of CBSI in the range of its etiology, therapy, and its origin, requires a multidisciplinary approach beyond routine assays and strategies. Precise diagnostics of *C. orthopsilosis* and other emerging *Candida* genospecies remains challenging. *C. orthopsilosis* should be considered in CBSI epidemiology. There is an urgent need for clear recommendations regarding *C. orthopsilosis* drug susceptibility breakpoints and further treatment. Genetic similarity of all *C. orthopsilosis* isolates, identified in our patient and highly related to type 1 of the genospecies, suggests catheter origin of the bloodstream infection. Long-term vascular catheter management must consider the risk of fungal transmission and adaptation. This should focus on the emerging genospecies detection, potential of strain selective pressure by antifungals–mediated therapy, and biofilm-producing activity of the genospecies.

## Figures and Tables

**Table 1:** Clinical and diagnosis data of 59-year-old male patient with CBSI caused by *C. orthopsilosis*.

Week of ICU Stay	1	2	3	4	5	6
Days of ICU stay	1–7	8–14	15–21	22–28	29–35	36–40
	Candida score components					
Clinical sepsis—2 points No clinical sepsis—0 point	0	0	0	0	0	0
Abdominal surgery—1 point No surgery—0 point	1	1	1	1	1	1
Total parenteral nutrition—1 point No total parenteral nutrition—0 point	1	1	1	1	1	1
Multifocal Candida colonization—1 point	0	1	1	0	0	0
Candida score (CS)—total points	2	3	3	2	2	2
	Laboratory parameters of infection					
CRP average (normal range, <5 mg/dL)	232	436/513	399	230	226	-
Procalcitonin (normal range 0.5–2 ng/ml)	0.6	0.7/1.03	0.6	0.5	0.5	0.5
WBC (in thousands/per cubic millimetre) (normal range, 4000–12,000/mm^3^)	29,000	29,000	27,000	15,000	12,000	8000
Fever 38 °C in days per week	1	2	4	6	2	2
Antifungals						
Fluconazole prophylaxis/therapy —days of ICU stay	all	none	none	none	none	none
Micafungin therapy —days of ICU stay	none	all	all	all	all	all
		Culture result				
Blood culture	negative	positive for *C. orthopsilosis* from 13th day of ICU stay				
*C. orthopsilosis* colonization	negative	positive (nasal swab)	negative from 3rd to 6th weeks of ICU stay			
*C. parapsilosis* colonization	positive skin swab on ICU admission	negative from 2nd to 6th weeks of ICU stay				
Fungal cell wall biomarkers						
1,3 β-D-glucan (pg/mL) in blood serum	<80	606.0	505.1	350.1	234.1	212.3
mannan (pg/mL) in blood serum	<62.5	<62.5	<62.5	<62.5	<62.5	<62.5

**Table 2:** Susceptibility of *C. orthopsilosis* and *C. parapsilosis* to antifungal drugs detected by E-test.

Day of ICU Stay	Name of Isolate	Source Sample	MICs of Antifungal Drug (µg/mL)				
			AMB *	FCA *	CAS *	MYC *	AND *
1	*C. parapsilosis* 37-1/17u	underarm skin	0.03	0.38	0.38	0.25	0.50
1	*C. parapsilosis* 37-2/17u	groin skin	0.03	0.38	0.38	0.25	0.50
13	*C. orthopsilosis* 80/17u	blood	0.25	0.19	0.50	0.25	0.50
13	*C. orthopsilosis* 88/17u	central line catheter	0.25	0.19	0.50	1.00	0.25
13	*C. orthopsilosis* 89/17u	blood collected by catheter	0.25	0.19	0.50	0.75	0.25
13	*C. orthopsilosis* 58/17u	nasal swab	0.03	0.50	0.25	0.25	0.25
16	*C. orthopsilosis* 38/17u	blood	0.25	0.50	0.25	0.25	0.50
20	*C. orthopsilosis* 57/17u	blood	0.25	0.50	0.25	0.50	0.25
40	*C. orthopsilosis* 3/17u	blood	0.25	0.50	0.25	0.50	0.25
*C. parapsilosis* ATCC 22019 (CBS604)		Reference	0.25	0.50	0.50	0.125	0.25
*C. orthopsilosis* ATCC 96141 (MCO456)		Reference (blood)	0.50	0.50	0.25	0.125	0.25

* AMB—amphotericin B; FCA—fluconazole; CAS—caspofungin; MYC—micafungin; AND—anidulafungin.

**Table 3:** Comparative analysis of ITS similarity between isolates derived from patient and GenBank, based on the neighbour-joining method.

Species	Name of Isolate	GenBank Accession No	Closest Relatives	GenBank Accession No	ITS Similarity %
*Candida parapsilosis sensu stricto*	37-1/17u	OQ253409	*C. parapsilosis* ZA039 *C. parapsilosis* ATCC 90018 *C. parapsilosis* ATCC 22019 *C. parapsilosis* AUMC 10197 *C. parapsilosis* AUMC 10215 *C. parapsilosis* AUMC 10243 *C. parapsilosis* SNPLPY2 *C. parapsilosis* IFM 62315 *C. parapsilosis* AUMC 10219	FJ662416 EU564206NNR130673 KU176110 KU200443 KX011602 MK859898L C390149 KU255845	100 99.81 99.81 100 100 100 99.81 99.81 99.81
	37-2/17u	OQ253410			
*Candida orthopsilosis*	58/17u	OQ253411	*C. orthopsilosis* ATCC 96139T *C. orthopsilosis* LEM071 *C. orthopsilosis* LMICRO175 *C. orthopsilosis* ATCC 96141 *C. orthopsilosis* JY 9 *C. orthopsilosis* IFM 49015	AJ698048 MG599183 KJ451699 EU564208 KM014573 LC389311	100 99.80 99.80 99.80 99.80 99.80
	88/17u	OQ253412			
	89/17u	OQ253413			
	80/17u	OQ253414			
	3/17u	OQ253415			

## Data Availability

All relevant data and materials have been included in the manuscript. Anonymous medical data used and/or analyzed during this study are available upon reasonable request and with the consent of the hospital.

## Supplementary Materials

The following supporting information can be downloaded at: https://www.mdpi.com/article/10.3390/ijms24076541/s1.
