# Supplementary figures and images for: Molecular Investigation of the Fatal Bloodstream Candida orthopsilosis Infection Case following Gastrectomy
Source: Int J Mol Sci. 2023 Mar 31;24(7):6541. doi: 10.3390/ijms24076541 (PMC10094972; doi:10.3390/ijms24076541)

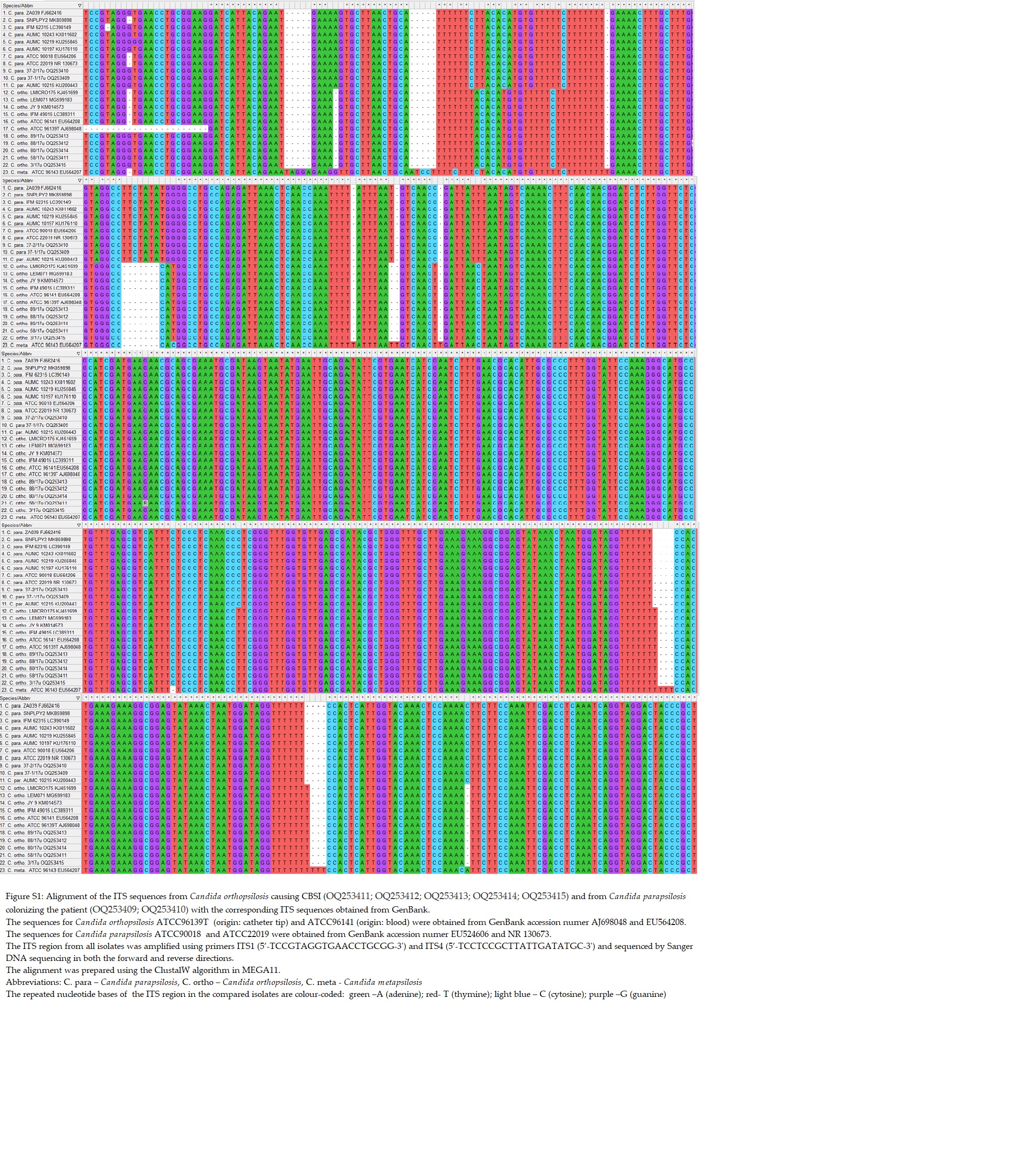

Supplement: Supplementary file 1 [file ijms-24-06541-s001.zip › ijms-2294385-supplementary/Figure S1.jpg]
